# The impact of pandemics: revising the Spanish Flu in Italy in light of models’ predictions, and some lessons for the Covid-19 pandemic

**DOI:** 10.1007/s40812-021-00182-1

**Published:** 2021-02-12

**Authors:** Enrico Berbenni, Stefano Colombo

**Affiliations:** 1 Department of Modern and Contemporary History, Università Cattolica del Sacro Cuore, Largo A. Gemelli 1, 20123 Milan, Italy; 2 Department of Economics and Finance, Università Cattolica del Sacro Cuore, Largo A. Gemelli 1, 20123 Milan, Italy

**Keywords:** Pandemic, Spanish flu, Italy, I10, N30

## Abstract

We consider some descriptive analysis of the main short- and medium-term economic indicators in Italy in the aftermath of the Spanish Flu pandemic. We analyse them in the light of the main neoclassical macroeconomic models of pandemics. Since most of the existing economic models about the consequences of a pandemic deal the pandemic event merely as a negative labour supply shock, we observe that some predictions of the theory about the economic impact of a pandemic seem not to be confirmed in the case of the Spanish Flu in Italy. In particular, economic indicators in Italy in the upsurge of the Spanish Flu need to be explained also in light of the consequences of the First World War. We use this evidence to discuss the predictions on the effects of the Covid-19 pandemic. We stress the importance of putting the analysis of the economic consequences of the current pandemic into the appropriate historical context.

## Introduction

The current COVID pandemic has induced several economists to try to predict the economic consequences of such a sudden global health crisis. This is an important challenge for the profession. Indeed, a correct assessment of the consequences of the pandemic could help to design the most appropriate economic measures to face the pandemic during the crisis and in the aftermath.

But how predicting the economic consequences of a pandemic? This is not an easy task. Typically, economists build on classic macroeconomic models and (try to) derive clear-cut implications of the shock caused by the pandemic in terms of variables such as wages, interest rates, consumption, inflation, GDP, and others (see Jordà et al. 2020, among the others).^1^

When the economic and financial crisis hit the world in 2008–2009, many scholars tried to compare that crisis with the 1929 Great Depression. Similarly, the current COVID-19 outbreak has generated great interest in the 1918 Spanish Flu. Indeed, many scholars believe that learning about the Spanish Flu short- and medium-term economic consequences might be useful to characterize the economic effects of the current coronavirus outbreak.

In this paper, we also make use of the Spanish Flu pandemic, and we discuss the implications of the Spanish Flu in Italy in the light of some simple macroeconomic models, e.g., the class of models used in Jordà et al. (2020), Karlsson et al. (2014), and Boucekkine et al. (2008), among the others, which are also used to make predictions about the effect of the current COVID-19 crisis.

In the present work, we focus on the historical evidence on the economic effects of the Spanish Flu epidemic in Italy, and we use this evidence to discuss the predictions on the effects of the Covid-19 pandemic. In particular, we provide some descriptive analyses about the short- and medium-term economic impact of the Spanish Flu in Italy, which seem to suggest that the economic impact of the Spanish Flu in the short- and medium-term, if any, doesn’t entirely fit the predictions of the economic theory. In contrast, in most of the cases, the economic performance in Italy under the Spanish Flu has been the opposite of what predicted by the standard macroeconomic models which are used to forecast the consequences of the current COVID-19 pandemic. One tentative explanation is that the Spanish Flu invested Italy and the rest of the world immediately after the end of the First World War, thus making very hard distinguishing the genuine effects of the pandemic from those of the war. In this sense, our considerations about the short- and medium-term impact of the Spanish Flu in Italy suggest putting the analysis of the economic consequences of any pandemic—included the current one—into the appropriate historical context in order to get a whole picture of the interconnected forces at work.

The rest of the paper proceeds as follows. In Sect. 2 we describe the economic consequences of a pandemic as derived by using standard neoclassic macroeconomic models. In Sect. 3 we briefly describe the Spanish Flu pandemic, with a particular focus on Italy. In Sect. 4 we test the main predictions of the macroeconomic models introduced in Sect. 2 by considering the Italian situation in the aftermath of the Spanish Flu and providing some descriptive analysis to assess the short- and medium-term impact of the Spanish Flu. Section 5 discusses and concludes.

## The economic impact of pandemics

In general, little is known about the economic consequences of a pandemic. However, the outbreak of COVID-19 makes it urgent to predict its economic impact in order to suggest the policymakers the correct economic measures to adopt. One possibility to assess the impact of pandemics consists in looking back to past pandemics and investigating their economic effects (see for example Brainerd and Siegler 2003). The underlying idea is that, given the similarities between pandemics in terms of worldwide diffusion and the great number of deaths, the impact of the current pandemic might be similar to the impact of previous pandemics. However, this kind of research is quite scant among economists. One understandable reason is the lack of administrative data for past pandemics (Meltzer et al. 1999). For example, the most deadly pandemic in the history, the Black Death in XIV century, that killed between one- and two-thirds of the European population according to Benedictow (2004), had certainly great economic and social consequences, but it is still uncertain the nature and the direction of these effects, due to the scarcity of the available data (see Munro 2009, and the references therein).^2^ Furthermore, due to the global nature of a pandemic, the effects spread across whole economies, for at least two reasons: the first is that the infection is widespread, and the second is that market integration diffuses the economic effects of the pandemic all over the world, thus making the disentanglement of all the effects extremely complicated. Therefore, no clear lessons can be easily derived from the observation of the economic impact of past pandemics.

An alternative approach consists in predicting the impact of pandemics starting from very general economic models and deriving endogenously the economic implications based on the common characteristics of all pandemics. This approach is highly appreciated by economists, as it does not rely on data (which are hard to obtain, especially for old pandemic phenomena), but rather on deductive reasoning. A prominent example of this approach is the following quote from the influential paper by Boucekkine et al. (2008): “*the whole story* [of a pandemic] *could be in principle reduced to an “initial condition shock*” (p. 8). Therefore, what consequences of pandemics are predicted by traditional neoclassic macroeconomic models?

Following Jordà et al. (2020), the common trait of large pandemics is described by the great number of human victims.^3^ In terms of a standard macroeconomic model *á* *la* Solow-Swan, the consequences of the pandemic are captured by “*the disproportionate effects on the labor force relative to land (and later capital)*” (Jordà et al. 2020, p.8). A similar view is shared, for example, by Karlsson et al. (2014), that claim that “[a pandemic] *represents a negative shock to labour supply, which on the other hand leaves physical capital intact*” (p.2). The European Commission (2006), when forecasting the economic effects of a pandemic, assumes “*a permanent negative shock to the population level […] This is the fundamental supply shock associated with the pandemic*” (p. 8). Garret (2009), quite explicitly, claims that “*the general conceptual foundation* […] *is that, ceteris paribus*, [a pandemic] *results in a large number of deaths which constituted a significant negative shock to manufacturing labour supply and thereby would have increased wages in the manufacturing sector immediately following* [the event]” (p. 712). In other words, neoclassic macroeconomic models interpret the pandemic mainly as a supply-side shock.

Both Jordà et al. (2020) and Karlsson et al. (2014) are streamlined versions of the complex model proposed by Boucekkine et al. (2008).^,^^4^,^5^ Boucekkine et al. (2008) build an economic model of pandemics and show that, in an exogenous growth model* á* *la* Solow-Swan with a concave production function, the short-run effect of the pandemic is a mechanical increase of per-capita income with respect to the equilibrium steady-state followed by a return to the initial steady-state through a recession.^6^ Indeed, suppose the epidemic takes place in a very short period of time, and after that the economy turns back to its initial epidemiological environment. In other words, the epidemic is like an “initial condition shock”. The pandemic kills part of the labour force, and this increases the capital per head compared to the initial steady-state level. It follows that the short-term effect of the pandemic is an increase of the income per head with respect to the equilibrium level. If the fertility behaviours do not change because of the pandemic,^7^ the population growth rate does not change. In this case, there is an excess of capital, and because of the decreasing returns of capital accumulation, the movement back to the initial steady-state of the economy is characterized by a recession (see Fig. 1 in Boucekkine et al. 2008).

When the growth is endogenously determined by the stock of human capital and absent technological progress, Boucekkine et al. (2008) show that after the pandemic less human capital is assigned to the education sector (because education is more intensive in human capital, and human capital is more costly now due to the higher wages induced by the relative scarcity of labour), and this has a negative impact on economic growth. Therefore, we should observe high wages and slow or negative growth.

Eichenbaum et al. (2020) emphasize the link between the pandemic evolution and the economic decisions of individuals, thus integrating the so-called SIR epidemiological models with micro-founded economic decisions by rational agents.^8^ Focusing mainly on the supply side, Eichenbaum et al. (2020) argue that people reduce their labour supply in order to limit their exposure to the risk of being infected. This supply-side consequence of the pandemic suggests a persistent recession (see also the calibrated model of McKibbin and Fernando, 2020). The macroeconomic SIR models also emphasize the existence of a trade-off between the mortality risk and the economic risk, which can be summarised as follows. During a pandemic, there is an increasing relation between the GDP level and the mortality risk, described by an increasing line, because working implies having contacts with other people, thus increasing the risk of being infected and perishing.^9^ In order to reduce the mortality risk, one has to accept a reduction of the GDP (for example, by imposing lockdowns). At the opposite, if one wants to increase the GDP level (for example, by removing lockdowns), an increase of the mortality cannot be avoided.

Guerrieri et al. (2020) posit a somewhat different question: what about the demand effects *induced* by the supply shock caused by the pandemic? Their argument could be summarised as follows. When workers lose their income due to the pandemic (for instance, because the firms are temporarily closed or go into bankruptcy) they lower their spending, thus making demand fall. It is shown that, under certain conditions, the demand shock (caused by the supply shock) can overcome the supply shock, thus provoking a demand-deficient recession, followed by a subsequent phase where real wages go up.

When focusing on *pure* demand-side implications of a pandemic, a number of implications can be considered. As emphasized by Baker et al. (2020), the patterns of consumers spending are likely to be hugely modified during a pandemic. Individuals want to reduce the possibility to be exposed to the virus, and this reduces the demand for goods and services that require having close contacts with other persons. This has an immediate and negative effect on aggregate consumption. Even if this negative effect could be partially compensated by stockpiling behaviours (especially at the outbreak of the pandemic, see Baker et al. 2020) and by the shift toward other goods (for example, healthcare items instead of restaurants and travels), the overall effect of a pandemic on aggregate consumption is likely to be negative (see for example the empirical exercise in Congressional Budget Office 2006). Indeed, the negative effect on aggregate consumption is magnified by lower income and/or higher income insecurity (due to unemployment) and by lower wealth (due to the reduction of the asset prices) (Muellbauer 2020). With regard to the other components of the aggregate demand, investments also reduce, due to wait-and-see delays by firms and lower investment opportunities because of the sluggish demand (Baldwin and Weder di Mauro 2020). For the same reasons, export and imports are expected to fall during a pandemic, but the net effect is ambiguous (Baldwin and Weder di Mauro 2020).^10^

Note that these models focus mainly on the short-term effects of a pandemic. When considering the long-term effects of a health crisis, a large amount of literature, recently summarized by Acemoglu and Johnson (2007), suggests that mortal diseases and pandemics have a long-term positive economic impact because, by contracting the labour supply and, consequently, increasing real wages, improve living conditions in the long term. This view is typically corroborated by the experience of the Black Death and its long-term impact on the European economy. For example, Herlihy (1997) argues that “*the Black Death gave to Europeans the chance to rebuild their society along much different lines [...] the unprecedented drain of the labor force [...] drove the need to produce labor-saving devices, and thus broke the stalemate of [the] feudal society [...] In the long run the late middle ages were a period of impressive technological achievement*”. Somehow similarly, Acemoglu and Robinson (2012) argue that an extreme event as the Black Death can disrupt the existing structure of the society opening the way for better long-lasting living conditions. In particular, they claim that “[a pandemic] *can open the way for breaking the cycle of extractive institutions and enable more inclusive ones to emerge*” (p. 101). Alfani and Murphy (2007) argue that “*there is evidence of a long-lasting improvement in European and Mediterranean real wages immediately after the Black Death*” (p. 330).^11^

At the opposite, a growing economic literature focuses on the long-term impact of pandemics through the effects on human capital accumulation. For example, Almond (2006) argues that a pandemic tends to reduce the educational attainments, thus decreasing human capital with negative effects on long-term GDP growth. In particular, the widely-mentioned article by Almond (2006) claims that a pandemic represents a shock hitting the capability of children to accumulate human capital with long-term consequences, and he finds evidence of underperformance of children born around 1918 (the year where the Spanish Flu spreads all over the world) in terms of education or income, and that this underperformance can be attributed to the pandemic episode. Indeed, according to Almond (2006) “*influenza infection of pregnant mothers caused the health of the cohort *in utero* to deteriorate as well. For example, the oxygen supply to the fetus may have been diminished by influenza or a secondary pneumonia infection*” (p.681). Other studies (see Black et al. 2007, and the references therein) have shown that fetal health is positively correlated with adult outcomes, thus yielding Almond (2006) to argue that the Spanish Flu, by lowering fetal health, hit future human capital accumulation of that cohorts.^12^ On the same line, Lin and Liu (2014) emphasize the long-term negative consequences of in-utero exposure to an epidemic disease by investigating the impact of the Spanish Flu on Taiwanese children born in 1918, and they find that this cohort is more likely to have permanent health problems, with a negative impact for the economic growth.

Aassve et al. (2020) point out that pandemics have long-lasting effects on individual behaviour by affecting social trust, that is, the general trust toward the others.^13^ In particular, the risk of being infected induces individuals to reduce social contacts, by avoiding large gatherings and interaction with unknown people. Besides, repeated suggestions by the authorities to reduce or avoid inter-personal contacts create suspicion among people. This profoundly lowers the level of trust in society. Notably, the authors empirically find that, in the case of the Spanish Flu, the mistrust generated by the pandemic had permanent consequences on individuals and it has been inherited to some degree by descendants. Since social trust is unambiguously recognized as an important factor for long-run economic growth (see for example Tabellini 2010), a pandemic, by permanently inducing mistrust and suspicion, is expected to have long-run negative economic effects.^14^ Another dimension of trust which is likely to be affected by the pandemic is the trust of citizens toward the political institutions, like leaders and political parties. Here the question is even more complex. Indeed, while social trust has unambiguously a positive effect on the well-being of a society, the trust in the political institutions during a pandemic might have ambiguous implications. Indeed, while some trust might promote good behaviours by leaders, too much confidence might induce citizens to believe that the government is effectively managing the pandemic even when it is not, thus inducing citizens to adopt bad behaviours and, by doing so, postponing the end of the pandemic and/or aggravating its social and economic costs (Levine et al. 2020).

Summarising, economic theory is quite ambiguous in its predictions about the long-term economic impact of a pandemic, whereas it is quite univocal with regard to the short- and medium-term consequences, claiming an increase of real wages of remaining workers and a reduction of GDP. In particular, neoclassical supply-side growth models focusing on short- and medium-term effects of a pandemic, suggest that, since the capital-labour ratio increases due to the pandemic, one should expect a pandemic to push up the real wages of the remaining workers.^,^^15^,^16^ Furthermore, in the short- and medium-term, a pandemic, by depressing the investments determines a negative shock to the aggregate supply, with negative effects on incomes and consumption, which end up in a downward shift of the aggregate demand, thus further reducing GDP (see for example Barro et al. 2020).^17^

To sum up, the main short- and medium-term economic implications of a pandemic as derived from standard neoclassical macroeconomics models are: a labour supply shock that induces an increase of the real wages, small or even negative growth rates of the economy, and a reduction of private consumption and investment.^18^, ^19^

## The Spanish Flu in Italy

In 1918 the world experienced the Great Influenza Pandemic, which is popularly known as the Spanish Flu, even if it was first identified in Kansas, US (Taubenberger 2006). The pandemic was caused by an H1N1 virus, that was identified only in 1933. The Spanish Flu has been by large the most devastating pandemic in human history in terms of the overall number of victims (and it is just second after the Black Death with regard to the number of victims over the number of existing people). It has been estimated that about 500 million individuals worldwide have been infected by the virus (Barro et al. 2020). The estimated number of deaths directly caused by the flu has been somewhere between 50 and 100 million people, in a period between 1918 and 1920 (Johnson and Mueller 2002). Almost all countries have been hit by the pandemic, with no significant difference between countries that participated in the war or remained neutral, and the mortality rate varied greatly across countries.^20^

The first wave of the Spanish Flu hit the European continent during the spring of 1918, probably carried by US soldiers during the First World War. After that, the spread of the flu was accelerated by the troop movements in the continent. The second wave of the Spanish Flu occurred between October 1918 and February 1919 and caused the greatest number of deaths. There has been also a third and a fourth wave of the pandemic, but they provoked a smaller number of victims. Therefore, the mortality rate of the Spanish Flu has been exceptionally high during the second wave. To put the Spanish Flu mortality in a context, it has been argued that during a normal influenza epidemic the mortality rate is about 0.1%, which means that one individual over 1000 infected people perishes. At the opposite, it has been estimated that the mortality rate of the Spanish Flu during the second wave has been about 2%, which means that one individual over 50 infected people perished (Erkoreka 2010, but Barro et al. 2020, suggest an even larger mortality rate). One of the peculiarities of the Spanish Flu was that it principally affected men and women between 15 and 44 years of age. While similar influenza diseases are characterized by a U-shape mortality distribution over age groups (thus killing mainly children and elderly people), the Spanish Flu had a W-shaped distribution over age (thus affecting mainly young individuals) (Erkoreka 2010; Kolata 2020). According to Barry (2017), this was because young adults have the strongest immune system, thus generating an excessive response to the virus attack. In other words, young people were not killed by the virus itself, but by the excessive reaction to the virus.

The airborne characteristic of the virus made it very difficult to contrast its diffusion. This created a lot of panic, mainly exacerbated by the repeated requests from governments to avoid interpersonal contacts in order to limit the diffusion of the virus. As reported by Barry (2005), in the rural areas of Kentucky, the Red Cross documented “*people starving to death not from lack of food but because the [healthy] were panic stricken and would not go near the sick*”. Furthermore, the panic was exacerbated by the fact that the world was not equipped to deal with the virus. No vaccine was available at that time, and any sort of cure (including colloidal mercury and alcohol) was suggested even if the benefits were unreliable. Therefore, local authorities attempted to limit the circulation of the virus by imposing quarantine, closing bars, cinemas and public entertainments, and preventing the entry into the community from the outside.^21^

When considering Italy, the Spanish Flu caused an impressive number of victims, even if compared with other similar countries. It has been estimated (Tognotti 2015), that about 600,000 individuals died in Italy due to the Spanish Flu, and that the mortality rate has been one of the highest in Europe. For example, Barro et al. (2020) document that the Spanish Flu death rate in Italy was 1.17% of the total population in 1918, which in Europe was lower only to Portugal (1.72) and Russia (1.42) (see Barro et al. 2020, Table 1).^22^

It is still not clear why Italy was so heavily affected. Even if other scholars (see for example Percoco, 2016) report lower estimates, there is a large consensus that the mortality in 1918 in Italy has been much higher than in the previous years even when taking into account the deaths caused by the war, thus highlighting the dramatic impact of the Spanish Flu (Table 1). As for the rest of the world, the pandemic lasted in Italy for the period 1918–1920, with a peak in 1918, which coincides with the last year of the war. In Italy, the heaviest incidence has been in the south, in particular in Sardinia, Calabria and Basilicata (Percoco 2016). The dramatic lethality of the flu in 1918 is documented by Boldrini et al. (1930), reporting that in Palermo 177 deaths were registered on 25 September (against a daily number of deaths around 20); in Naples 256 deaths were registered on 7 October (against a daily number of deaths around 40); in Rome 256 deaths were registered on 21 October (against a daily number of deaths around 30).

**Table 1:** Total deaths in Italy, 1910–1930 (,000)

1910	698
1911	760
1912	651
1913	679
1914	656
1915	821
1916	866
1917	959
1918	1.281
1919	685
1920	690
1921	650
1922	699
1923	663
1924	671
1925	678
1926	689
1927	648
1928	653
1929	675
1930	583

Source: Istat, Popolazione residente e bilancio demografico ai confini dell'epoca—Anni 1862–1947 (http://seriestoriche.istat.it/, last accessed: 25 May 2020)

As for the other countries, young people seemed to be more susceptible to the flu. As reported by Erkoreka (2010), 35% of the total deaths in Rome caused by the flu in 1920 were in the 20–40 age range, followed by the > 60 age range (27.5% of total deaths) and by the 0–5 age range (13.3% of total deaths), thus showing typical W-shaped distribution of lethality over age.

However, a peculiar characteristic of the impact of the Spanish Flu in Italy is the high mortality among young women. Some authors (Boldrini et al. 1930; Pinnelli and Mancini 1999) argue that this might be due to the fact that young women usually worked at home and were frequently caregivers, thus having frequent contacts with sick and infected people. The impact of flu on sex mortality differentials emerges after the fifth birthday, according to Pinnelli and Mancini (1999). Indeed, according to Giannini (1931) and L’Eltore (1947), the different roles of boys and girls started to emerge after 5 years of age, thus accentuating the greater exposure of girls relative to boys to sources of infection.^23^

Overall, due to the combined effect of the pandemic and the war, the Italian population reduces from 37,023 million at the beginning of 1917 to 36,241 million at the end of 1919 (ISTAT). Several factors might have contributed to the diffusion of the flu in Italy as well as to the high mortality rate in the country. First, it could be noted that the peak of the flu coincided with the end of the First World War. Therefore, there was a great movement of troops and refugees in the Italian territory that contributed to the spread of the flu. As noticed by Pinnelli and Mancini (1999), “*the first days of victory coincided with the worst outbreaks of the epidemic, thus constituting a further source of risk for public health. Moreover, Italian soldiers began to return pell-mell from the front and prisoner of war camps, undernourished and worn-out ‘physically and morally from the treatment that had been meted out’ and thus an easy prey to infection and disease (*Mortara, 1925*)*”.

Second, the Spanish Flu not only overlapped with the end of the Great War but also with the resurgence of tuberculosis, a disease that has long been present in Italy and that, according to statistics, reaped more than 50,000 human lives every year. The conflict was not only a facilitating factor in the Spanish epidemic but had also serious consequences on the spread and lethality of tuberculosis. Indeed, by the end of 1915, the death toll of tuberculosis had already exceeded the average for the three years 1912–1914 by 5,000 units. In 1916 the deaths exceeded 60,000 units. In 1918, although excluding over 200 municipalities in the Veneto involved in military operations, mortality from tuberculosis reached 74,000 units, equal to 2.09‰, with an increase of 41.5% compared to 1912–1914. Furthermore, many deaths from tuberculosis were ascribed to the contextual outbreak of Spanish Flu (Detti 1984). Therefore, the concurrence of multiple events (the war and the tuberculosis disease) represented a major concern in the management of public health, thus making it very difficult for Italy to face the upsurge of the Spanish Flu.

Finally, the living conditions of the Italian population at that time have been an aggravating factor of the disease. Most people were living without running water, electricity, and toilets, and overcrowding in the houses was normal, thus reducing the health of individuals and then increasing the lethality of the flu. As reported by Martini et al. (2019) some health measures have been taken to reduce the number of contacts between people (for example, the advice to maintain distance between people), even if there is no evidence that commercial and productive activities have systematically been closed as with the lockdown during the current COVID-19 pandemic. For instance, Tognotti (2015) reports that many of the non-pharmaceutical interventions not only were too mild and ineffective, but they also were introduced only when the spread of the flu was out of control. In addition to this, the health system was weakened by the fact that many doctors and health operators were at the war front "*thus weakening the defences of other regions and the efforts to protect the civil population from the epidemic*" (Mortara, 1925).

## Main economic indicators in Italy in the aftermath of the Spanish Flu

In Sect. 2, we have identified four sets of effects that result from pandemics, i.e.: (1) supply-side effects (falls in labour supply and increases in real wages), (2) demand-side effects (falls in consumption), (3) human capital effects (poorer health and education), and (4) social trust effects (between individuals, and towards political institutions). Leaving aside the latter—due to insufficient data and the need to address the issue by means of in-depth research—we try to observe what the historical experience of the Spanish Flu in Italy tells us about the above-mentioned variables. We also add the effects on inflation and public finances.

Broadly speaking, we know little about the short- and medium-term consequences of the Spanish Flu on the Italian economy. The impact of the war overlapped with that of the pandemic, making it difficult to distinguish the exact causes. In other words, there is the risk of attributing to the pandemic effects that are largely the consequence of the war. Therefore, the predictions of economic theory should be interpreted in light of the historical context.

First, did the Spanish Flu *really* represent a shock to the labour supply? As illustrated in Sect. 2, neoclassic macroeconomic models mainly interpret a pandemic as a negative shock to labour supply. Though this may have been true because of the young age of most of the victims, in practice the issue appears more complicated. The war had already resulted in a reduction in the labour supply of six million young people called to the army: 680.000 soldiers died and 463.000 were disabled or mutilated. During the conflict, they were partially replaced by women and minors, while the organization of factory work was subject to structural changes, being subordinated to the overriding war production needs. Social legislation about working hours was often disregarded. Agricultural production, on the other hand, was only partially affected by the labour shortage, due to the widespread underemployment in the countryside and to larger resorting to the female workforce (Bof 2009). Therefore, a negative effect of the flu on labour supply, if any, has not probably been dramatic, even in 1918.^24^ Furthermore, at the end of the war, the demobilization of the army put almost three million men on the job market in less than two years. It follows that, during 1919, the return of soldiers from the front caused an inverse shock to the labour supply, which exceeded demand by far. For example, it is interesting to observe that the Bank of Italy attributed the rarefaction of labour in 1918 and early 1919 *only* to the war mobilization, *without any explicit reference to the pandemic* (Banca d’Italia 1919, p. 18–19).^25^ Anyhow, among the main causes of troubles for some primary industrial sectors, such as the cotton and iron and steel industry, there seemed to be a concomitance of factors that were not limited to the workforce alone, such as the volatility of the exchange rate, the high freight rates and the shortage of raw materials and fuel. This scarcity was caused by the weak recovery of international trade and by the serious losses suffered by the Italian merchant navy during the war.

Second, did the Spanish Flu induce a real wage rise, as one would expect following an increase in the capital-labour ratio? Indeed, growth models *á* *la* Solow-Swan predict that, because of the shortage of labour, the short-run effect of the pandemic is an increase of the per-capita income compared to the equilibrium steady-state. It can be observed that the upsurge in real wages was noticeable—especially factory workers and day labourers—but this was not the case in 1918. Indeed, such increase occurred in 1919 and, for some categories, in 1920 (Table 2), that is when there was not a shortage of manpower (see our discussion above). The wage rise was rather the consequence of violent social requests in 1919 and 1920, aiming at recovering the purchasing power eroded by inflation both in the rural and in the industrial sectors (Fabbri 2009; Cotula and Spaventa 2003).

**Table 2:** Real wages, 1913 = 100 (1913–1920)

	Day labourers	Factory workers
1913	100	100
1914	103	102
1915	103	103
1916	109	92
1917	113	88
1918	107	79
1919	130	109
1920	118	137

Source: Zamagni, 1993, p. 308

**Fig. 1:**
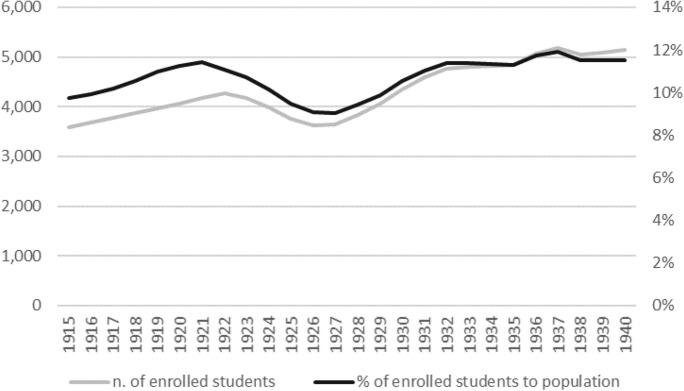
- Enrolled students in primary school: total number (thousands, left axis), per cent to population (right axis). Source: Istat, Iscritti a scuola o all'università per livello di istruzione, sesso e anno scolastico o accademico—Anni 1861/62–2013/14 (http://seriestoriche.istat.it/, last accessed: 17 November 2020)

Third, did the pandemic negatively affect human capital? As argued in Sect. 2, a pandemic negatively affects human capital through both short-term and long-term channels. From a short term perspective, less human capital is assigned to the education sector during a pandemic (see Boucekkine et al. 2008), whereas, from a long-term perspective, a pandemic hit the capability of children to accumulate human capital (see Almond 2006). As a first approximation, the trend of primary school enrollments shows a lasting decrease from 1923 to 1926 and then starts to grow again. Figure 1 may reflect the demographic shock of 1918–1919 and the subsequent population increase, but its progression allows also other considerations.

Following the seminal study carried out on the United States by Almond (2006), Percoco (2016) focuses on the long-run consequences of Spanish Flu exposure in terms of human capital accumulation in Italy, and finds a small but persisting negative effect—an average reduction of 0.3–0.4 years of schooling for the cohort born in 1918–1920—on the rate of accumulation of human capital. This was also likely to reflect on regional productivity. One outstanding result of the analysis is that the impact of the influenza was heterogeneous across space, provided the very different mortality rates across the Italian regions with the Southern ones being the most affected. To put it differently, “*children born during the influenza pandemic received less schooling than the following and preceding cohorts, and the magnitude of such effects depended on a measure of child exposure during the first four years of life, as well as on the region of birth*” (Percoco 2016, p. 1498). In any case, the different impact of the pandemic across the country must be interpreted in the light of a strong and persistent long-term regional divide in human capital accumulation. In this regard, Cappelli (2015) underlines how the primary school organization of post-unification Italy had led to a human capital trap. The convergence in education attainments remained incomplete in the interwar period, despite the public policy's effort (see the Daneo-Credaro reform of 1911) to bridge the gap by replacing the current decentralized system of school funding by municipalities with a centralized system. Against this backdrop, therefore, the Spanish Flu seems to have contributed, albeit marginally, to slowing down an already complex process of convergence.

Fourth, did the influenza reduce consumption and investments? Indeed, both consumption and investments are expected to decrease both because of pure-demand effects (people reduce consumption in order to avoid interaction with other persons, see Baker et al. 2020) and as a consequence of a supply shock (since income is reduced, people reduce consumption and investments, see Guerrieri et al. 2020). Concerning private consumption, historical statistics (Table 3) do not seem to support this hypothesis, or at least they do not indicate that the pandemic had a markedly depressing effect. Private consumption was continuously decreasing since the beginning of the war, as typically occurs during wartime, and consequently, it started to rise again in 1919. Within this framework, food accounted for 60% of total consumption between 1916 and 1920, on average. It is interesting to note that, despite the fall in domestic production during the war, the decrease in food availability was limited by resorting to imports, especially from the United States.^26^ Public consumption, on the other hand, moved in the opposite direction, as one would expect given the growth of state orders during the war and their subsequent contraction at the end of hostilities. The decrease in total consumption between 1919 and 1921 was therefore brought about by the sudden reduction of the public demand and not of the private one. As for investments, a constant declining trend is recorded in every category throughout the war, but they started rising already in 1919, showing no significant connection to the Spanish Flu outbreak (Table 4).

**Table 3:** Private and public consumption (1938 prices, historical boundaries), million lire (1913–1920)

	Private consumption	Public consumption	Total consumption
1913	94,648	9,381	104,029
1914	82,764	14,174	96,938
1915	62,578	37,028	99,606
1916	53,083	61,717	114,799
1917	38,288	79,237	117,526
1918	33,020	83,577	116,597
1919	50,524	56,003	106,527
1920	76,785	29,320	106,105

Source: Istat, Serie storiche della contabilità nazionale (1861–2017) (http://seriestoriche.istat.it/, last accessed: 25 May 2020)

**Table 4:** Investments (1938 prices, historical boundaries), million lire (1913–1920)

	Plant, machinery, and transport equipment	Constructions	Other investments	Total Investments
1913	6788	5466	2260	14,514
1914	6068	5785	2018	13,871
1915	3946	4260	1091	9296
1916	3655	2369	541	6565
1917	3318	1562	486	5366
1918	2445	1371	206	4023
1919	3529	3006	323	6858
1920	6327	3311	931	10,568

Source: Istat, Serie storiche della contabilità nazionale (1861–2017) (http://seriestoriche.istat.it/, last accessed: 25 May 2020)

Fifth, did the flu significantly affect the GDP of 1918 and the early post-war years, as expected by standard neoclassic macroeconomic models of pandemics (see for instance Jordà et al. 2020; Karlsson et al. 2014; Boucekkine et al. 2008)? To explain the contraction of GDP in 1918 and 1919 (chained values, Table 5), we must consider the interplay of several variables. Both historical-economic literature and contemporary sources highlight the difficult compromise between social aspirations and the reorganization of the Italian industry and public finance as one of the main drivers behind the post-war sluggish economy. These complications resulted in the crisis of both big industrial companies (as Ilva and Ansaldo) and large banks (as Banca Italiana di Sconto and Banco di Roma) (see for example Einaudi 1933; Toniolo 2003; Cafaro 2014).

**Table 5:** Italian GDP at market prices, chained values and GDP per capita (1913–1925)

Year	GDP at market prices—million euros (chained values; reference year = 2010)	GDP at market prices—million euros (chained values; reference year = 2010) Annual rate of change (%)	GDP per capita—thousands of euros (chained values; reference year = 2010)
1913	126,144.52	5.21	3.39
1914	119,313.24	− 5.42	3.20
1915	114,970.59	− 3.64	3.04
1916	125,657.70	9.30	3.29
1917	125,895.89	0.19	3.30
1918	121,847.55	− 3.22	3.22
1919	114,960.56	− 5.65	3.09
1920	118,046.81	2.68	3.16
1921	114,596.76	− 2.92	3.06
1922	124,273.30	8.44	3.28
1923	135,825.57	9.30	3.55
1924	139,512.55	2.71	3.61
1925	149,138.08	6.90	3.83

Source: Istat, Serie storiche della contabilità nazionale (1861–2017) (http://seriestoriche.istat.it/, last accessed: 25 May 2020)

The industrial adaptation from war to peace production was one of the major concerns, after years in which the Italian industry had benefited from large public contracts and high profits: war expenses—which may be classified as public consumption (see also Table 3)—as a percentage of GDP had reached 33.1% in 1917 and 1918, compared to 18.3% in 1915 (Zamagni, 1993). Besides, wartime industrialization had been structurally unbalanced, favouring some productive sectors and being located almost exclusively in the north-western area of the country. Especially large-scale industry had taken the opportunity to grow through horizontal and vertical integrations, while the end of the war led to a sudden excess of production capacity and an increase in unemployment. At the end of the conflict, this expansion called for a radical reorganization of the industrial system, a goal which, however, was not easy to achieve.

Furthermore, the return of soldiers from the front caused widespread discontent both in the countryside and in factories, and a strong increase in social conflict due to the promises received during the war for an improvement in living conditions. The growth of inequalities was one major legacy of the conflict, which negatively affected not only the working and peasant classes but also the middle ones, whose wages could not keep up with the high inflation. Strikes, occupations of lands and factories were, therefore, numerous during 1919 and 1920 (the so-called “Biennio rosso”). A keen observer like Luigi Einaudi stressed the importance of social instability in preventing postwar economic recovery (Einaudi 1919a, b, c).

The precarious conditions of public finance were another factor of weakness, due to the upsurge in public debt, which increased from 74% of GDP in 1914 to 160% in 1920 (Zamagni 1998b). Especially foreign debt settlement was a key issue for securing public finances in the aftermath of the war. It follows that the leeway for an expansive public policy was necessarily limited, thus constituting another serious constraint on the recovery of the Italian economy.

Finally, the monetary disorder following the war made the general situation even worse. Although inflation did not reach the levels of Germany and Austria, it did not stop in the post-war period but continued to grow significantly until 1920, mostly due to the expansion of the state's financial needs and the worsening of its budget deficit. The high inflation (Table 6) was fuelled by the growth in paper circulation (from 2.7 billion lire in 1914 to 11.9 in 1918, up to 19.8 in 1920) which, together with debt, had been the primary instrument of war financing, leading to the abandonment of gold standard. The increased monetary circulation accounted more than product shortage for the soaring prices and the high volatility of exchange rates at the end of the war (Bof 2009; Zamagni 1993; Banca d'Italia 1920). Again, a direct connection to the pandemic cannot be detected, while economic theory predicts ambiguous consequences on inflation due to the opposite effects on prices of a decrease of both supply and demand.

**Table 6:** Wholesale prices and cost of living (index numbers)

Year	Wholesale prices	Cost of living
1914	100	100
1915	133	107
1916	193	134
1917	286	189
1918	431	264
1919	470	268
1920	617	352
1921	565	417
1922	569	414

Source: Istat, 1966

Summarising, the short- and medium-term economic consequences of the Spanish Flu could be confused with the consequences of the First World War and the other historical events that occurred in Italy at that time, thus making it difficult if not impossible to distinguish them. For example, the destruction of fixed (along with human) capital in north-eastern Italy due to the war cannot be ignored. Likewise, after the defeat of the Italian army in Caporetto (October 1917) and until the Italian counter-offensive in October 1918, part of those territories remained under Austrian occupation. The trend of the national GDP largely reflects these events.

The data presented in the tables above show that a complex interplay of variables was in place in the aftermath of WWI, which profoundly altered the most immediate impact of the pandemic on GDP and other variables. Of course, following counterfactual reasoning, one could ask what would have happened in normal market conditions, predicting a much more adverse effect of the Spanish Flu on the Italian economic system. According to a cross-country comparative study carried out by Barro et al. (2020), the epidemic is estimated to have reduced real per capita GDP by 6.0 per cent in the typical country. However, a deviation from this estimate is likely to be very high due to interfering variables, as we have argued above.

## Discussion and conclusions

The emergence of the COVID-19 pandemic has generated a growing stream of contributions aiming to suggest correct policy measures to face the current crisis and the aftermath of the pandemic. In order to assess the economic consequences of a pandemic, general neoclassical macroeconomic models are often used. In most of the economic models dealing with the consequences of a pandemic, this event is merely reduced to a negative labour supply shock (see for example Boucekkine et al. 2008; European Commission 2006; Karlsson et al. 2014; Jordà et al. 2020; Garrett 2009, among the others). It follows that the short- and medium-term consequences of the outbreak are easily derived, and they can be summarised by a temporary increase of the real wages, a slow or negative growth rate of the GDP, and a reduction of private consumption and investment.^27^

In this paper, we focus on the consequences of the Spanish Flu pandemic in Italy, and we discuss its economic implications in light of some common neoclassical supply-side growth models. In particular, we consider some descriptive analysis of the main economic indicators in Italy during and after the Spanish Flu upsurge. The indicators put the predictions of the economic theory into a more complex interpretative framework. Indeed, our analysis, even if purely descriptive, seems to suggest that the Italian economic performance during the Spanish Flu is somewhat different from what predicted by the economic theory. In particular, the *Great Influenza* in Italy can hardly be considered a dramatic labour supply shock if one takes into account the simultaneous demobilization of the army that increases the number of job-seekers by almost three million men. Consequently, the rise of real wages, that occurred in 1919–1920, cannot be interpreted as the consequence of the increase of capital-labour ratio, but rather as the consequence of violent social requests in that period. Similarly, private consumption did not significantly reduce during the Spanish Flu pandemic, but it rather moderately increased because of the end of the war. Finally, even if the GDP decreased in the after-war period, historical explanations seem to be better suited to explain the phenomenon rather then referring to a quite disputable negative supply labour shock.^28^ In other words, the economic impact of the pandemic can be hardly distinguished from the consequences of the First World War.

With regard to the current COVID-19 pandemic, its economic impact is also likely to be affected by several historical factors that are not easy to incorporate into standard models.^29^ For example, it seems quite difficult to suppose that the capital-labour ratio could significantly increase as a consequence of the current pandemic when over the last few decades the participation in the market economy of China, India, and former communist countries has caused the doubling of the world's workforce (the "Great Doubling", according to the expression coined by Richard Freeman, see Freeman 2005). Furthermore, rather than modelling the COVID-19 pandemic as a supply labour shock, a correct assessment of the current crisis should recognize that “[as] *this virus is extremely contagious but not especially fatal […] the containment measures – the disruption to work processes, the limitations on meetings and travel – will be a larger negative supply shock than the number of deaths, even if the latter could still turn out to be large*” (Weder di Mauro, 2020, p.31).

Needless to say, there are also several differences between the current pandemic and the Spanish Flu pandemic, which make a direct comparison between the two pandemics hard to perform. First, in the time of the current crises, the demand shock driven by the behavioural changes of the consumers induced by the pandemic is likely to be stronger than during the Spanish Flu pandemic. Indeed, as argued by Wren-Lewis (2020), “social” consumption (i.e. consumption that requires having contact with other people – think for example to shopping at malls or travels) is stronger today than at the beginning of the XX Century. Furthermore, the tourism industry is much more relevant for the world economy today than during the Spanish Flu pandemic. The current pandemic imposed relevant restrictions to people mobility, thus dramatically hitting the tourism industry (Sigala 2020). For all these reasons, the demand-side effect of the COVID-19 is likely to be stronger than the demand-side effect of the Spanish Flu.

Second, globalisation has a multiplicative effect on the current crisis. For example, consider the wait-and-see attitude of consumers. The widespread diffusion of the information about the pandemic is likely to amplify the fear of people of being infected and suffering health and/or economic consequences of the pandemic, thus generating more prudential behaviour in consumption.^30^

Third, the world economy is much more interconnected today than at the beginning of the XX Century (Baldwin 2013). Therefore, an economic slowdown hitting one country as a consequence of the pandemic is likely to affect all the other countries through the global value chains (GVC), which represent formidable transmission channels of the economic contagion (Coveri et al. 2020).

Finally, mass vaccinations are possible in the current pandemic and not during the Spanish Flu. Mass vaccinations, while creating the expectation that the pandemic will be ended in a reasonable time, posit a tremendous logistic challenge that humanity probably never experienced before. It is not clear whether and how the priority given to the vaccination of the population would affect the economy.^31^

Of course, many other issues have not been considered in the present paper. Within this framework, they may prove crucial to assess correctly the consequences of a pandemic, today as well as a hundred years ago. First, the asymmetric impact of a pandemic within the population and across countries is likely to aggravate the existing inequalities.^32^ Second, the disruption in the global value chain caused by a pandemic might change the configuration of the world economy for a long period (Coveri et al. 2020; Strange 2020). Third and above all, “*in times of rising nationalism and populism, people's fears and suspicions of ‘others’ might become a force for disintegration and deglobalisation*” (Baldwin and Weder di Mauro 2020, p. 22). This applies today as in the aftermath of the First World War. Indeed, the pandemic may well accentuate the reasons for contrast that are already present on a global scale and forces countries to behave selfishly: borders have been shut down, reciprocal blames for the source of the virus emerged, and there have been diplomatic fights for healthcare supplies.^33^ One consequence of the virus diffusion today is that people accept to lose some rights (for example, free movement) in change for security: this increases the power of the States, which in turn pushes up the ideological identification of people with the State.^34^

## Footnotes

1 One reason for this is the urgency of providing suggestions: building on solid models allows saving time and reduces the risk of being misunderstood.

2 For a classical approach to pandemics in the pre-industrial Europe, see Cipolla (1974).

3 Indeed, Jordà et al. (2020) identify a pandemic as a global epidemic phenomenon that causes more than 100,000 victims.

4 Boucekkine et al. (2008) distinguish between short-term and long-term pandemics. A short-term pandemic lasts months or a few years; a long-term (or persistent) pandemic lasts decades. The Spanish flu is an example of short-term pandemic, whereas the HIV pandemic is an example of long-term pandemic. While writing, the fast development of an effective vaccine against the COVID-19 disease suggests considering the COVID-19 pandemic as a short-term one. In the text, we consider the implications of short-term pandemics.

5 It should be emphasized that, according to the authors, “*an epidemiological shock is modelled by an initial shortfall in the size of population*” (Boucekkine et al. 2008, p. 6), that is, the pandemic is mainly modelled as a negative shock to the labour supply.

6 The amount of saving can change in two directions as a consequence of the pandemic. On one hand, the fact that active people died, pushes the savings down. On the other hand, pandemic is often associated to increasing uncertainty which induces precautionary saving (the so called “wait-and-see” attitude of consumers), thus pushing the saving rate up. Furthermore, if the amount of capital decreases, the marginal productivity of capital increases and the real interest rate increases: this also induces the individuals to save more. Since this positive effect on saving is likely to prevail, one might expect a transition to a *new* steady state. Even in this case however, in the case of decreasing returns of capital, it can be shown that the transition phase is characterized by a recession (see Boucekkine et al. 2008, pp. 6–7).

7 In the case of short-term pandemics, fertility behaviours are likely not to change dramatically.

8 See Atkeson (2020) for an introduction and a review of SIR models, where SIR refers to “susceptible” (S), “infected” (I) and “recovered” (R) individuals.

9 At the opposite, during the pre-pandemic situation, there is a downward relationship between the GDP level and the risk of mortality. Indeed, when GDP increases, living conditions ameliorate and the risk of mortality reduces. We thank one reviewer for suggesting this example.

10 A somehow related question is the role played by the restrictions to the movement of people, goods, and services, on the economic impact of a pandemic. Clearly, such restrictions made it possible to contain the diffusion of the contagion (and the related human costs and indirect economic costs). However, at the same time, reducing mobility has a dramatic direct economic effect, as it limits the benefits of globalization. In a recent article, Voth (2020) posits the following question: how much mobility should a globalised world have? Since globalization increases the risk of a new pandemic, according to Voth (2020), in the next future humanity should consider the possibility to limit unnecessary mobility of people to prevent future outbreaks of new pathogens, while preserving free exchange of goods and capital, as very few diseases are transmitted by contaminated goods.

11 A similar conclusion with regard to the Italian economy can be found also in Malanima (2002).

12 See also Kelly (1950) for a similar result in the case of a severe influenza episode in UK in the 1950s. However, some authors (see for example Brown 2011) dispute the validity of these claims.

13 According to Almond and Verba (1963), there is social trust if for the majority of people “*most people can be trusted*”.

14 The disruption of social trust is one of the most-known consequences of all epidemics: “*One Citizen fled after another. Parents nor kindred never visiting them, but utterly they were forsaken on all sides*” (Boccaccio, *Decameron*, 1350).

15 For example, a consistent narrative suggests that the Black Death, by inducing labour scarcity, is one of the main causes of the increase of real wages in Europe in XIV and XV centuries (Munro 2009; Herlihy 1997).

16 Symmetrically, these models claim that the real natural rate of interest is likely to reduce over the short- and medium-term, due to the reduction of the investment demand induced by the scarcity of labour. In other words, greater returns to labour go hand-in-hand with lower returns to capital.

17 The effect on inflation is ambiguous.

18 In Sect. 4, we will test these short- and medium-term predictions by looking at the Spanish Flu pandemic in Italy. In our analysis, we do not consider the long-term economic impact of the Spanish Flu, apart from human capital for the reasons that we will make clear below. This for two reasons: on one hand, as argued above, economic theory is quite ambiguous in its predictions about the long-term impact of a pandemic; second, sufficient temporal observations are often not available, so that it is very difficult to understand the complementary effects of the pandemic over the long run.

19 Needless to say, this kind of predictions strongly depends on the theory the models refer to. The vast majority of the papers discussing the economic impact of the pandemic adhere to the so-called neoclassic approach (see Boucekkine et al. 2008, and the other papers mentioned in Sect. 2), which dates back to the well-known Solow-Swan model (Solow 1956; Swan 1956). Clearly, when adopting a different theoretical framework, the implications of a pandemic could be dramatically different. Consider for example the standard Kaldor-Pasinetti growth model (Pasinetti 1962), rooted in the Post-Keynesian tradition. Within this theoretical set-up, the pandemic is expected to determine a reduction in both investments and consumption. As the equilibrium growth rate is endogenously determined by the investments (see also Garegnani 1992), the pandemic is expected to lower the growth rate, and by doing so, also the wages, thus contrasting with the predictions of neoclassic growth models.

20 As reported by Barro et al. (2020), the highest cumulative death rate was for Kenya, followed by India, Guatemala and Madagascar. However, according to Tomkins (1992), the greatest burden of the Spanish Flu was carried by the island of Samoa, where one person over four was killed by the flu.

21 An interesting description of the non-pharmaceutical intervention in US during the Spanish Flu can be found in Markel et al. (2007).

22 However, there is not consensus about the ranking. For example, according to Collier (1982), cited by Tognotti (2015), “*Italy* *recorded 10.6 deaths per 1000 inhabitants, the highest mortality rate in Europe, occupying the first position in the world after India, Madagascar, Mexico, Africa, New Zealand, Guatemala, Eastern India and* *Chile*”.

23 A strong difference in the mortality rates of the Spanish Flu between males and females can be observed also in Paris during 1918 (Erkoreka 2010). However, “*the large difference between the male and female data is due to the fact that young men were engaged in fighting at the war fronts, explaining why the number of dead women is much higher*” (Erkoreka 2010, p. 84).

24 See also Karlsson et al. (2014) for a similar argument relative to Sweden in the aftermath of the Spanish Flu.

25 Similarly, with regard to the conditions of agriculture in 1919, the Bank of Italy's annual report stated: “*As a whole, the agricultural production of 1919 was poor, notably due to the dominant drought in almost all regions of Italy. In part, the state of workforce deficiency was also responsible for this, a deficiency that gradually disappeared in the second half of the year following the dismissal of the army*” (Banca d'Italia, 1920, p. 27–28).

26 Instead, during the Second World War, the average intake of calories fell sharply (Zamagni 1998a, pp. 182–185).

27 In Sect. 2 we stressed that alternative models – namely Post-Keynesian macroeconomic models – could provide different predictions of the impact of a pandemic. However, the ongoing academic discussion about the effects of the current COVID-19 pandemic is dominated by standard neoclassical growth models.

28 Notably, our cautionary conclusion is in line with the results of Karlsson et al. (2014), that study the impact of the Spanish Flu on the Sweden economy. They find some evidence that the capital returns have been negatively affected by the pandemic, but, contrary to the theoretical predictions, they find no evidence of the impact of the pandemic on real wages.

29 The main similarity between the Spanish Flu and the current COVID-19 pandemic can be identified in the airborne nature of the virus. Given this characteristic, under both pandemics the social interaction between people has been deeply limited, thus having similar effects in terms of disruption of social trust (see Sect. 2).

30 This was clear during the subprime crisis in 2008–2009. While few nations have been directly touched by the subprime crisis generated in the US, the psychological shock induced many firms to postpone investments and many consumers to postpone purchases (Baldwin and Weder di Mauro 2020).

31 Needless to say, the current pandemic is likely to affect countries to different extents. As argued by the European Commission (2020), Italy is likely to be the most hit in terms of GDP decrease among European countries (the estimated GDP downturn is 11.2% in 2020). According to Sapir (2020), the differences between countries in terms of the economic hit from COVID-19 can be mainly explained by the lockdown strictness, the quality of governance, and the share of tourism in the economy.

32 As noted in Sect. 3, differently from the Spanish Flu pandemic (whose impact was W-shaped relative to the age), the COVID-19 pandemic affects mainly older adults. As argued by Li and Mutchler (2020) with regard to the COVID-19 pandemic consequences in US, this yields disproportionate challenges for older adults. In particular, older people “*who are coping with high living expenses […] are simultaneously exposed to higher rates of infection*” (Li and Mutchler 2020, p. 479). In other words, more economically vulnerable people are even more vulnerable to the consequences of the virus, thus posing a relevant challenge for social securities systems (pensions, health systems…).

33 For example, at the end of February, a truck filled with medical equipments has been stopped at the German-Swiss border by the German authorities.

34 As noted by Fiedler and Tagespiegel (2020), crisis are likely to increase cohesion within countries while often generate hostility between them. The rising of nationalism after the First World War is often interpreted by historians as a response to the social insecurity generated by the war.
